# Apoptotic Protease Activating Factor-1 Inhibitor Mitigates Myocardial Ischemia Injury via Disturbing Procaspase-9 Recruitment by Apaf-1

**DOI:** 10.1155/2017/9747296

**Published:** 2017-11-27

**Authors:** Ying Wang, Qiuyan Zhang, Liangjie Zhong, Mingshen Lin, Xiaoling Luo, Siyu Liu, Peng Xu, Xinhua Liu, Yi Zhun Zhu

**Affiliations:** ^1^Department of Pharmacology, School of Pharmacy, Fudan University, Shanghai 201203, China; ^2^TA Instruments-Waters LLC, Shanghai, China; ^3^School of Pharmacy, Macau University of Science and Technology, Macau

## Abstract

(2S,3S,4S,5R,6R)-6-(4-((4-guanidinobutoxy)carbonyl)-2,6-dihydroxyphenoxy)-3,4,5-trihydroxytetrahydro-2H-pyran-2-carboxylic acid (ZYZ-488) was discovered as a novel inhibitor of apoptotic protease activating factor-1 (Apaf-1). In present work, a surface plasmon resonance (SPR) assay confirms the direct binding between ZYZ-488 and Apaf-1 and this interaction was found to be able to block the recruitment of procaspase-9 by Apaf-1. This study also shows that the treatment of MI (myocardial infarction) mice with this novel Apaf-1 inhibitor remarkably reduces the infarct size, improves cardiac functions, and attenuates the histopathology changes caused by MI. Meanwhile, here it is shown that ZYZ-488 decreases myocardial enzyme release, inhibits cardiomyocyte apoptosis, and suppresses the activation of the downstream cascade of caspases. Moreover, in silico prediction validated the drug-like properties of ZYZ-488. In conclusion, our findings present the first piece of evidence indicating the interaction between Apaf-1 and procaspase-9 as a novel therapeutic target in myocardial infarction and suggesting ZYZ-488 as a promising therapeutic option for myocardial infarction disease.

## 1. Introduction

Myocardial infarction is still the most common cardiovascular disease and a leading cause of worldwide death [1]. Acute myocardial infarction is a fatal and acute disease of the cardiovascular system that threatens human health [2].

A variety of animal and human studies have demonstrated that apoptosis contributes significantly to cardiomyocyte loss during the development and progression of heart disease [3]. Myocardial apoptosis is a key pathologic feature of acute myocardial infarction and heart failure [4]. Promoting cell survival by inhibiting apoptosis is one of the available strategies to attenuate cardiac dysfunction caused by cardiomyocyte loss. Overcoming hypoxia-induced cardiac apoptosis, however, remains challenging for the treatment of various heart diseases [5]. Apoptotic protease activating factor-1 (Apaf-1), the central component of the apoptosome, is subjected to major conformational changes during mitochondrial apoptosis [6]. The apoptosome recruits and activates an initiator member of the caspase family of cysteine aspartyl proteases, procaspase-9, that in turn activates apoptosis-effector caspases initiating therefore apoptotic cell death [7]. In our previous work, we synthesized a novel compound ZYZ-488 which exhibited significant cardioprotective property *in vitro* and ZYZ-488 was demonstrated a novel inhibitor of Apaf-1. The chemical structure of ZYZ-488 and its parent drug LEO can be seen in our previous study. *In vitro* study of ZYZ-488 suggests that ZYZ-488 as a potential inhibitor of Apaf-1 elicited a significant cardioprotective effect on hypoxia-induced cardiomyocytes. As the first molecule reported to reduce cardiomyocyte apoptosis by targeting Apaf-1, the potential of ZYZ-488 for treating myocardial infarction *in vivo* is unknown. In addition, our previous study showed that ZYZ-488 significantly attenuated the activation of procaspase-9 and procaspase-3, while the inhibition effect was dependent on the levels of Apaf-1 in the cell [8]. Even though, the direct binding between Apaf-1 and ZYZ-488 and the concrete mechanism still need to be further investigated.

In this study, we used surface plasmon resonance analysis (SPR) to investigate the binding activity of ZYZ-488 to Apaf-1. It provides detailed information on binding affinity, the association and dissociation kinetics of the interacting partner. Importantly, the interaction is monitored in real time [9, 10]. This powerful, label-free technique is commonly used to measure the molecular interactions of small molecules with their biological targets like proteins and DNA. Moreover, we elucidated the cardioprotective effect of ZYZ-488 in mice with myocardial infraction and the involved mechanisms. Then considering druggability predictions are important to avoid intractable targets and to focus drug discovery efforts on sites offering better prospects [11]. Drug-like properties of ZYZ-488 as a potential candidate for myocardial infraction was evaluated through in silico predictions by ADMET Predictor™ software.

## 2. Investigations and Results

### 2.1. ZYZ-488 Binds Directly towards Apaf-1 and Then Blocked Procaspase-9 Recruitment

**Figure 1:**
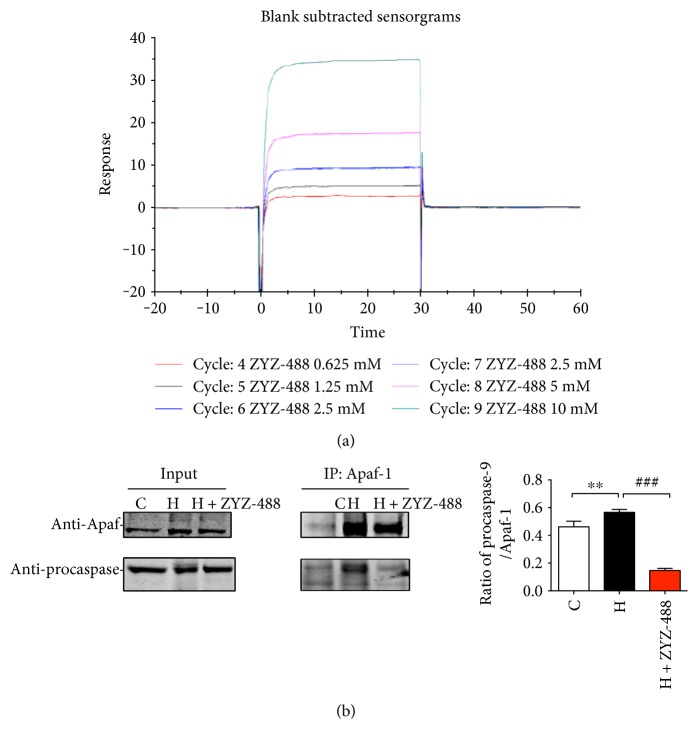
Interaction analysis of Apaf-1 in binding with ZYZ-488 and procaspase-9. (a) Kinetic analysis of binding behavior between ZYZ-488 and Apaf-1. The *y*-axis presents the amount of bound ZYZ-488 as relative response (RelResp (RU)), and the *x*-axis presents time (s). Various concentrations of ZYZ-488 were used. (b) The anti-Apaf-1 antibody was applied for precipitating extracts of H9c2 cells and coimmunoprecipitation of caspase-9 was revealed. The coimmunoprecipitation of Apaf-1 with caspase-9 was confirmed using Apaf-1 and caspase-9 antibodies. C: control group; H: hypoxia group; H + ZYZ-488: hypoxia with ZYZ-488 treatment group. H9c2 cells were treated with ZYZ-488 in hypoxia for 12 h. Data were presented as mean ± SEM. ^∗∗^*P* < 0.001 versus control; ^###^*P* < 0.001 versus hypoxia.

The chemical structure of ZYZ-488 and LEO can be seen in our previous study [8]. *In vitro* study of ZYZ-488 suggests ZYZ-488 as a potential inhibitor of Apaf-1-elicited significant cardioprotective effect on hypoxia-induced cardiomyocytes [6]. Here, the binding ability of ZYZ-488 to Apaf-1 was determined by surface plasmon resonance (SPR). SPR is a cell-free system for detailed study of biomolecular interactions. The binding affinity of ZYZ-488 to Apaf-1 was reflected by response unite (RU) values. The curve of cycle 6 was basically coincidence with the cycle 7 curves. This suggests the good reproducibility in the experiments. As Figure 1(a) showed, the absorption response (AbsResp (RU)) increased apparently following the ZYZ-488 injection which confirmed the direct interaction between ZYZ-488 to Apaf-1.  Table 1 displayed the kinetics parameters data. Relative response (RelResp (RU)) of each cycle was calculated by the AbsResp minus its baseline response unite. RelResp increased with the lifting of ZYZ-488's concentrations in a dose-dependent manner (Table 1). This indicated that ZYZ-488 bound to the Apaf-1-immobilized surface in a dose-dependent manner. Besides, the kinetic curves showed a rapid association and dissociation behavior. Also, the slopes inferred that ZYZ-488 has a fast binding speed to Apaf-1.

During apoptosis, cytochrome *c* induces the oligomerization of Apaf-1 in the presence of *d*ATP or ATP, forming the so-called “apoptosome,” and then the apoptosome recruits and facilitates the proteolytic processing of procaspase-9 [12]. To explore whether the direct binding of ZYZ-488 towards Apaf-1 could disturb the recruitment of caspase-9 by Apaf-1, coimmunoprecipitation between caspase-9 and Apaf-1 was performed. As shown in Figure 1(b), Apaf-1 recruited and interacted with much caspase-9 in hypoxia-induced apoptosis. However, ZYZ-488 treatment strongly inhibited the coimmunoprecipitation of caspase-9 compared with hypoxia group. These results indicate that ZYZ-488 could directly bind with Apaf-1 then inhibited Apaf-1's recruitment of caspase-9.

### 2.2. ZYZ-488 Elevated Cardiac Function after MI Surgery

**Figure 2:**
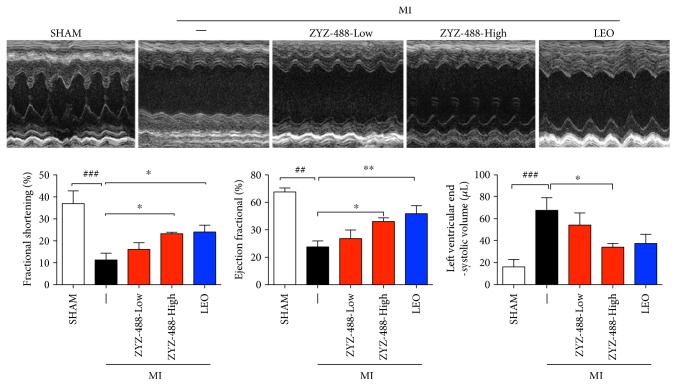
ZYZ-488 improved cardiac function of MI mice. Representative echocardiographic graphs and quantification of ejection fraction (EF%), fractional shortening (FS%), and left ventricular end-systolic volume (LVESV). ZYZ-488-Low: myocardial infarction with ZYZ-488 (33.9 mg/kg); ZYZ-488-High: myocardial infarction with ZYZ-488 (67.8 mg/kg); LEO: myocardial infarction with LEO (43.2 mg/kg). Data were presented as mean ± SEM. ^##^*P* < 0.01, ^###^*P* < 0.001 versus SHAM; ^∗^*P* < 0.05, ^∗∗^*P* < 0.01 versus MI.

To test the in vivo efficacy of ZYZ-488, we tested its effect on an established MI model using ligation of left coronary artery operation. The mice were intramuscularly administrated with ZYZ-488 (33.9 mg/kg or 67.8 mg/kg) or LEO (43.2 mg/kg), and LEO were used as positive control. Cardiac function was examined using echocardiography at 72 hours after. Compared with SHAM group, MI mice showed much lower eject fraction (EF) (27.48 ± 4.47% versus 67.58 ± 3.13%; *P* < 0.01) and fractional shortening (FS) (11.25 ± 2.56% versus 36.93 ± 2.39%; *P* < 0.001), whereas left ventricular end-systolic volume (LVESV) were increased significantly (66.83 ± 12.18% versus 15.97 ± 2.77%; *P* < 0.001) indicating impaired cardiac function (Figure 2). As illustrated in Figure 2, ZYZ-488 (67.8 mg/kg) significantly increased EF (46.13 ± 4.59% versus 27.48 ± 4.47%) and reduced the LVED (34.00 ± 3.44% versus 66.83 ± 12.18%). These results demonstrated that ZYZ-488 could reduce the enlarged volume of left ventricle caused by MI as well as decrease the thickness of ventricular wall. ZYZ-488 preserved the cardiac function in a dose-dependent manner.

### 2.3. Attenuation of Myocardial Infarction Injury by ZYZ-488

**Figure 3:**
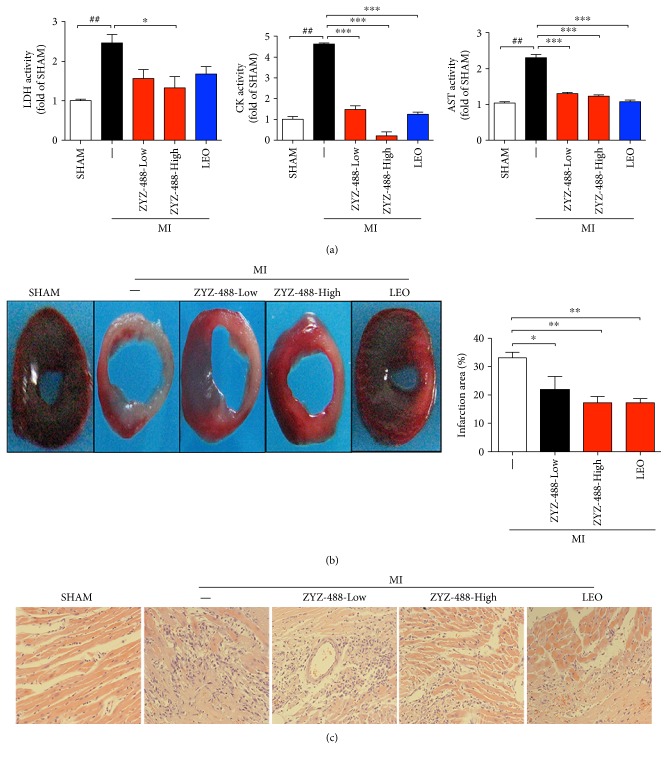
ZYZ-488 alleviated acute myocardial infarction injury. (a) Quantification of LDH, CK, and AST content in mice serum. (b) Infarct size of mice heart was determined by triphenyltetrazolium chloride (TTC) staining. (c) Histological analysis with H&E staining on heart sections. Representative microphotography of H&E stained sections from border zone in indicated groups. ZYZ-488-Low: myocardial infarction with ZYZ-488 (33.9 mg/kg); ZYZ-488-High: myocardial infarction with ZYZ-488 (67.8 mg/kg); LEO: myocardial infarction with LEO (43.2 mg/kg). Data were presented as mean ± SEM. ^##^*P* < 0.01 versus SHAM; ^∗^*P* < 0.05, ^∗∗^*P* < 0.01, and ^∗∗∗^*P* < 0.001 versus MI.

The cardioprotective effects of ZYZ-488 were verified *in vivo* at an acute phase of mice myocardial infarction. Lactic dehydrogenase (LDH), creatine kinase (CK), and aspartate aminotransferase (AST) are three reliable biomarkers of cardiomyocyte injury [13]. To confirm the degree of cardiomyocyte injury in ischemia, we determined LDH, CK, and AST concentrations in serum.  Figure 3(a) showed that LDH, CK, and AST concentrations in serum were markedly increased in the MI group compared with the SHAM group (^##^*P* < 0.01). However, treatment with ZYZ-488 effectively decreased the serum level of LDH, CK, and AST in a dose-dependent manner (33.9 mg/kg and 67.8 mg/kg). Then, triphenyltetrazolium chloride tissue enzyme staining technique (TTC) was applied for early-phase acute myocardial infarct size quantification. Comparing the MI group (33.15 ± 1.62%), LEO (17.44 ± 1.14%), low (21.89 ± 4.07%), and high (17.33 ± 2.45%) dose of ZYZ-488 treatment reduced myocardial infarction size (*P* < 0.05) (Figure 3(b)). ZYZ-488 elicited similar potent alleviating myocardial infarction with its parent drug LEO (*P* > 0.05). Subsequently, hematoxylin and eosin (HE) staining was performed to depict the histopathology in mice heart after MI. Myocardial cells of SHAM group were arranged in neat rows with no inflammatory cell infiltration as showed in Figure 3(c). Compared with SHAM group, the number of myocardial cells in the infarction zone was reduced with more inflammatory infiltration. At the same time, the myocardial fibers ruptured, dissolved, and arranged disorderly (Figure 3(c)). With either high dose ZYZ-488 or LEO treatment, the HE-stained slide of these two groups showed remarkably reduced degree of inflammation and myocardial cell necrosis. Consistently, myocardial cells of ZYZ-488 group (67.8 mg/kg) and LEO group were arranged more narrowly, suggesting that ZYZ-488 protected hearts against acute myocardial infarction.

### 2.4. ZYZ-488 Protected against Myocardial Apoptosis

**Figure 4:**
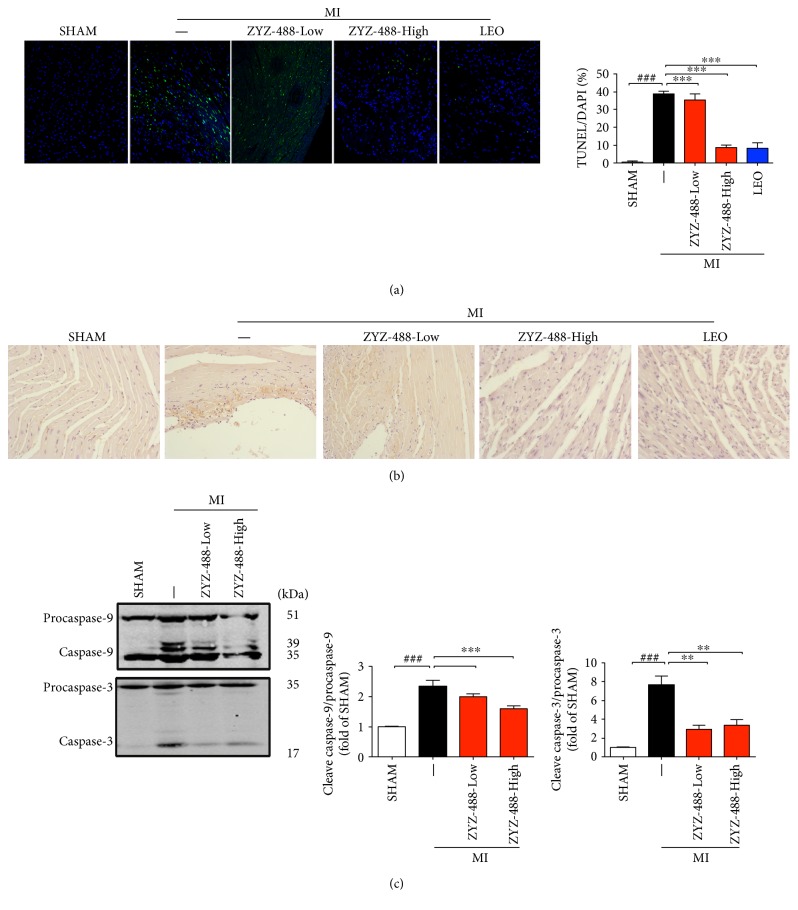
ZYZ-488 protects the heart against apoptosis after MI injury. (a) Representative images of TUNEL staining (green) and DAPI staining (blue) on hearts; (b) IHC staining for cleaved caspase-9 protein; (c) ZYZ-488 inhibited Apaf-1-mediated activation of procaspase-9 and procaspase-3. ZYZ-488-Low: myocardial infarction with ZYZ-488 (33.9 mg/kg); ZYZ-488-High: myocardial infarction with ZYZ-488 (67.8 mg/kg); LEO: myocardial infarction with LEO (43.2 mg/kg). ^###^*P* < 0.001 versus SHAM group, ^∗∗^*P* < 0.01; ^∗∗∗^*P* < 0.001 versus MI group. Data are the mean ± SEM of results from at least three independent experiments.

Apoptosis is recognized as a physiological counterpart of cell replication and is the contributing cause to cardiomyocyte cell death during ischemia, MI, and heart failure [14]. Here, we used TUNEL assay to determine the effects of ZYZ-488 in myocardial infarction-induced apoptosis (Figure 4(a)). After surgery, the number of positive cells increased (0.58 ± 0.023% versus 38.45 ± 1.76%) (*P* < 0.001), but significantly reduced with ZYZ-488 treatment at 67.8 mg/kg (8.65 ± 1.31% versus 38.45 ± 1.76%) (*P* < 0.01) (Figure 4(a)). Then, immunohistochemistry for cleaved caspase-9 revealed that treatment with ZYZ-488 decreased the cleaved caspase-9 levels induced by MI injury (Figure 4(b)).

In mitochondrial apoptosis pathway, cytochrome c and *d*ATP trigger the assembly of the Apaf1-apoptosome, which recruits and cleaves caspase-9 [15]. The WB results indicated that acute myocardial infarction caused by ligation of the left coronary artery induced an increase in cleaved caspase-9/procaspase-9 ratio (2.34 ± 0.17 versus 1.00) and cleaved caspase-3/procaspase-3 ratio (7.64 ± 0.77 versus 1.00) compared with SHAM group (Figure 4(c)). Consistent with the results *in vitro*, ZYZ-488 suppressed the cleavage ratio of caspase-9 [33.9 mg/kg (2.00 ± 0.07%versus 2.34 ± 0.17%), 67.8 mg/kg (1.59 ± 0.07% versus 2.34 ± 0.17%)] and the subsequent activation of caspase-3 [33.9 mg/kg (2.92 ± 0.36% versus 7.64 ± 0.77%), 67.8 mg/kg (3.38 ± 0.44% versus 7.64 ± 0.77%)] after myocardial infarction injury (*P* < 0.05). These results validated that ZYZ-488 inhibited the activation of procaspase-9 pathway.

### 2.5. Druggability Assay of ZYZ-488 (ADMET Predictions)

The computational approach, also known as in silico approach, is one of the modern and fastest developing techniques being used today in drug discovery to assess the pharmacokinetics, ADME (absorption, distribution, metabolism, and excretion), and toxicity predictions [16]. With the intention to find out and compare the drug-like properties for ZYZ-488 and LEO, ADME calculation was performed by ADMET predictor of Simulations Plus. An inspection of the data given in Table 2 reveals the molecular descriptors, such as molecular weight (MW), logP, hydrogen bond donor (HBD), and hydrogen bond acceptor (HBA). Either ZYZ-488 or LEO shows acceptable logP and logD parameters. ZYZ-488 shows good drug-like ability, including reasonable logP and logD, and predicted human jejunal permeability (Peff) for both compounds is greater than 0.1 cm/s × 104, which indicates their good membrane permeability. It may also be interpreted from Table 2 that ZYZ-488 will show better oral absorption than LEO due to its good solubility (11.4 mg/mL). Tissue distribution of a drug is another important consideration in drug development. Volume of distribution (*V*_d_), an important parameter, relates the amount of a drug in the body to the measured concentration in a relevant biological fluid [16]. Percent of drug unbound to plasma proteins (*F*_up_) is used to identify the binding of the inhibitors to the carrier protein in the blood. The blood to plasma ratio determines the concentration of the drug in whole blood compared to plasma and provides an indication of drug binding to erythrocytes. The prediction results of *V*_d_, RBP, and Fup suggest that the compound ZYZ-488 has a pretty good distribution quality.

Cytochrome P450 enzymes are responsible for most phase I metabolism of foreign compounds. Our current data suggests that compound ZYZ-488 is stable facing members of CYP450, while LEO was predicted to be the substrate of CYP3A4 (Table 3) and Cl_int_ was 0.246 (*μ*L/min/mg). Uridine diphosphoglucuronosyl transferase (UGT) catalyzes the conjunction between glucuronic acid and small molecule. This reaction is an important part of phase II metabolism. Only LEO was metabolized via two different isoforms of UGT which leads to poor bioavailability. This prediction is consistent with pharmacokinetics study of leonurine in rats [17] that leonurine was mainly metabolized *in vivo* by glucuronidation. These results predicted that the bioavailability of leonurine after oral administration may be very low. Compared with LEO, ZYZ-488 was predicted not to be metabolized by UGT enzymes. Considering this, ZYZ-488 has more preferable drug-like qualities.

## 3. Discussion

The Apaf-1 molecule is a Y-shaped protein with the molecular weight in the range of 133.3–141.8 kDa, composed of N-terminal caspase recruitment domain (CARD), a central nucleotide-binding oligomerization domain (NOD), and 12-13 repeats of WD40 (WRD) [18]. Apaf-1 as a key molecule in the mitochondrial pathway of apoptosis, which forms a large complex known as apoptosome, then recruits and activates procaspase-9. Besides, reports have emerged showing nonapoptotic functions for Apaf-1. It has been demonstrated that Apaf-1 is involved in the centrosomal microtubule nucleation process and cytoskeleton organization [19]. The absence of Apaf-1 leads to the dysregulation of centrosomal microtubule nucleation. Therefore, Apaf-1 has a crucial role in the formation of normal bipolar mitotic spindle, cell division, and cell migration which involve centrosome. This may indicates that excessive Apaf-1 inhibition might be deleterious. The Apaf-1 inhibition pattern and moderate potency are the key points for Apaf-1 inhibitor development and application. Malet et al. [20] identified a novel class of trialkylglycine-based molecules that bind reversibly to Apaf-1 in a cytochrome c noncompetitive manner. A chemical inhibitor of Apaf-1 named QM31 exerts mitochondria-protective functions while the interaction between QM31 and Apaf-1 was not deciphered [6]. Different from previous Apaf-1 inhibitors, the SPR assay demonstrated that ZYZ-488 binds directly to Apaf-1 then dissociates from Apaf-1. Then result of coimmunoprecipitation verified ZYZ-488's binding disturbed recruitment of procaspase-9 by Apaf-1. Those results suggest that ZYZ-488 act in a reversible binding to Apaf-1, and this may allow the recovery of the potential physiological role of Apaf-1 in the heart in not-stressful conditions. ZYZ-488 presents a novel class of Apaf-1 inhibitor which disturbs procaspase-9 recruitment by Apaf-1.

Inhibiting the apoptotic protease activating factor-1 pathway strikingly alleviates apoptosis [21, 22]. However, the study on therapeutic application of Apaf-1 inhibitors was fairly limited. Orzaez et al. demonstrated that SVT016426 avoid apoptosis in *in vivo* models of kidney ischemia [7]. They proposed that the SVT family of Apaf-1 inhibitors binds to Apaf-1 at the CARD-NOD interface or at the reported *d*ATP binding site in the NOD domain. Minocycline was found to inhibit cell death and decrease mutant Huntingtin aggregation by targeting Apaf-1 [23]. Here, our data further suggested treatment with Apaf-1 inhibitor: ZYZ-488 significantly reduced the infarct size, improved cardiac functions of MI mice, and attenuated the histopathology change caused by MI.

Investigations into the causes of late-stage failures in drug development, performed in the 1990s, revealed that poor pharmacokinetics and toxicity were often responsible [24, 25]. Drug-like properties of ZYZ-488 was evaluated through in silico predictions. We found that ZYZ-488 have more acceptable physiochemical parameters and biopharmaceutical qualities compared with its parent drug LEO, especially on the metabolism stability. Taken all, our present investigation firstly shed light on the beneficial role of recruitment of procaspase-9 inhibition in myocardial infarction. At the same time, ZYZ-488 represents a powerful therapeutic option for ischemic heart therapy.

## 4. Experimental

### 4.1. Cell Culture and Induction of Hypoxia

Hypoxia cell model was induced in H9c2 cells as previously reported [8]. H9c2 cells were treated with 10 *μ*M ZYZ-488 in hypoxia for 12 h.

### 4.2. SPR Biosensor Analysis

SPR biosensor analysis was used to verify the binding of ZYZ-488 to Apaf-1 protein, which was analyzed by SPR-based Biacore T200 instrument (GE Healthcare, Pittsburgh, PA, USA), as described previously. In brief, the protein was immobilized on a CM5 sensor chip in 4500 RU, since the Apaf-1 protein is large in MW and it is difficult to immobilize. ZYZ-488 was diluted to different concentrations with in running buffer, and it was then injected into the channels for protein binding at a flow rate of 10 mL/min, followed by washing with the running buffer. The analysis of the sensorgram curves obtained at different concentrations of ZYZ-488 use of BIA evaluation v4.1 (Biacore).

### 4.3. Animal Model

The experimental protocol was approved by the ethical committee and conformed to internationally accepted ethical standards. The animals were supplied by the Laboratory Animal Centre, Fudan University, Shanghai, China. Healthy male C57BL/6 mice (8–10 weeks old) were used for this experiment. Left descending coronary artery ligation was performed to induce MI model as previously reported [26]. All surgeries were performed under anesthesia of isoflurane.

### 4.4. Experimental Design and Drug Administration

Before surgery, mice weight 20–25 g were randomly divided into five groups: (1) SHAM group; (2) myocardial infarction group (MI); (3) myocardial infarction + ZYZ-488 (33.9 mg/kg); (4) myocardial infraction + ZYZ-488 (67.8 mg/kg); and (5) myocardial infarction + LEO (27.5 mg/kg) with 15 mice in each group. ZYZ-488 and LEO were administered intramuscularly immediately after surgery for 3 days.

### 4.5. Echocardiography

72 hours after the surgery, mice were anesthetized with isoflurane and placed on a heating pad. Cardiac function of mice was dynamically evaluated by echocardiography using Vevo 770 (Visual Sonics Inc., Toronto, Canada). The transducers with frequency of 10 MHz for ventricular structure provided spatial resolutions. The transducers with frequency of 17.5 MHz for ventricular structure provided spatial resolutions. Left ventricular internal dimension in systole (LVIDs), left ventricular internal dimension in diastole (LVID_d_), left ventricular anterior wall in systole (LVAWs), left ventricular anterior wall in diastole (LVAW_d_), left ventricular posterior wall in systole (LVPWs), and left ventricular posterior wall in diastole (LVPW_d_) were obtained from the M-mode tracings, while other parameters such as left ventricular volume in systole (LVs), left ventricular volume in diastole (LV_d_), ejection fraction (EF), and fractional shortening (FS) were obtained automatically by the high-resolution electrocardiograph system.

### 4.6. Triphenyltetrazolium Chloride (TTC) Staining

Infarct heart size was determined by 1% triphenyltetrazolium chloride (TTC) staining. After echocardiography assay, animals were sacrificed and hearts excised immediately, washed with cold PBS, then incubated in 1% (*w*/*v*) TTC (0.1 M PBS buffer) for 30 minutes at 37°C. Then, each heart was cut manually into six transverse slices. Infarct size was assessed as the ratio of viable and nonviable myocardium area using ImageJ software (NIH, Boston, MA) to analyze.

### 4.7. Detecting Myocardial Enzymes

72 hours after surgery, blood was collected and serum was separated by centrifugation at 2500 ×g for 5 minutes at 4°C. The myocardial enzymes (lactic dehydrogenase (LDH), creatine kinase (CK), and aspartate aminotransferase (AST)) were measured according to the manufacturer's instructions (Nanjing Jiancheng Bioengineering Institute, Nanjing, China).

### 4.8. Histological and Immunohistochemical Staining

Animals were sacrificed and the hearts were isolated and then fixed in 4% paraformaldehyde. Each heart was embedded in paraffin and cut into sections (5 *μ*m thickness). The sections were used for hematoxylin and eosin (HE) staining or immunohistochemical (IHC) staining. Histopathology of mice myocardium after myocardial infraction was evaluated by hematoxylin and eosin (HE) staining kit (YuanYe Biotech, Shanghai, China) according to the manufacturer's instructions. For IHC staining, the sections of heart tissue were deparaffinized, rehydrated with freshly deionized water, and washed thrice with phosphate-buffered saline (PBS). The sections were then incubated with primary antibodies overnight at 4°C (Cell Signaling Technology, USA). Sections were again washed with PBS and incubated with biotinylated secondary antibodies at 37°C for 30 min. After washing thrice with PBS for 5 minutes, the sections were incubated with 0.05% (*w*/*v*) 3,3′-diaminobenzidine tetrahydrochloride dihydrate, counterstained with hematoxylin, dehydrated, and mounted for microscopic detection.

### 4.9. Determination of Apoptosis by TUNEL Staining

Apoptosis in infarct area was determined with a TUNEL staining kit (Biotools Co., Shanghai, China) according to the instructions. Apoptotic ratio of the infarct zone was determined as number of TUNEL-positive cells to total number of nucleus stained. Six fields were included for each section and average number of three sections was used as percentage of apoptosis for each group.

### 4.10. Coimmunoprecipitation and Western Blot Assay

Coimmunoprecipitation was performed by using Santa Cruz Protein A/G Plus Agarose (sc-2003) as the datasheet instruction. Briefly, the anti-Apaf-1 antibody (Proteintech: catalog number 21710-1-AP) was applied for precipitating extracts of H9c2 cells, then collected the precipitation, then caspase-9 antibody (CST: 9508) was used to reveal the coimmunoprecipitation procaspase-9 and Apaf-1. Whole-cell protein extract was prepared as input. Cell lysates were separated by SDS-PAGE and transferred to NC membranes. After being blocked with 5% skim milk for 1 h at room temperature, membranes were incubated with primary antibodies against Caspase-9 (Cell Signaling Technology, Irvine, CA), followed by incubation with the specific secondary antibodies. The reactive bands were detected by infrared fluorescence and exposed to the Odyssey Analysis System (Gene Company Limited, USA).

### 4.11. ADMET Predictions

In silico ADME studies were performed using ADMET predictor of Simulations Plus (Simulations Plus™, Lancaster, CA, USA, http://www.simulationsplus.com) in which various pharmacokinetic parameters like logP, LogD, hydrogen bonding descriptors, polar surface area (PSA), human intestinal absorption, and plasma protein binding (PPB) were estimated for ZYZ-488 and LEO.

### 4.12. Statistical Analysis

Values are shown as means ± SEM of at least 3 independent preparations. The significance of differences between groups was evaluated with *t*-tests or one-way ANOVA followed by the Dunnett's multiple comparison tests. Values of *P* < 0.05 were considered significant. All statistics were carried out using PRISM 6.0 for MAC.

## Figures and Tables

**Table 1:** Kinetics parameters for the binding of ZYZ-488 to Apaf-1.

Concentrations (mM)	AbsResp (RU)	Slope (RU/s)	RelResp (RU)
0	6744.9	NO	0
0.625	6747.1	0.02678	3.9
1.25	6749.7	0.03706	6.2
2.5	6754.2	0.02754	10.5
5	6762.9	0.03689	18.7
10	6780.2	0.06737	35.9

**Table 2:** The physicochemical properties, ADMET predictions of compound. All descriptors are generated by the ADMET predictor software of Simulations Plus.

Property	Description	Recommended range	ZYZ-488	LEO
logP	Octanol-water partition coefficient	<4.5	−0.89	0.89
logD	Octanol-water distribution coefficient	<3.5	−0.89	−0.2
Peff	Human effective jejunal permeation in cm/s × 10^4^	≥0.1	0.18	0.32
*S*_w_	Aqueous solubility in mg/mL	≥0.010	11.4	6.06
*V*_d_	Volume of distribution in L/kg	≤3.7	0.51	1.05
RBP	Blood-to-plasma concentration ratio	<1.0	0.81	0.89
*F*_up_	Percent of drug unbound to plasma protein	>2%, <95%	41.86	38.23

**Table 3:** Prediction of various isoforms of phase I and phase II metabolism enzyme.

Compound	ZYZ-488	LEO	
Phase I	N	CYP3A4	Cl_int_ (*μ*L/min/mg) = 0.246
Phase II	N	UGT1A1, UGT2B7	
